# Supplementary material for: In vitro and in silico studies for the identification of anti-cancer and antibacterial peptides from camel milk protein hydrolysates
Source: PLoS One. 2023 Jul 12;18(7):e0288260. doi: 10.1371/journal.pone.0288260 (PMC10337890; doi:10.1371/journal.pone.0288260)
Supplement: S1 File — (DOCX) [file pone.0288260.s001.docx]

***In vitro and in silico studies for the identification of anti-cancer and antibacterial peptides from camel milk protein hydrolysates***

Mohammad Javad Taghipour, Fard Ardekani, Hamid Ezzatpanah*, Mohammad Ghahderijani

^1,2*^ Department of Food Science and Technology, Science and Research Branch, Islamic Azad University, Tehran, Iran

^3^Department of Agricultural Systems Engineering, Science and Research Branch, Islamic Azad University, Tehran, Iran

**Table S1. The peptides derived from *in silico* enzymatic digestion of the various proteins of camel milk**

| **Protein name** | **Enzyme name** | **Sequence of peptide** |
| --- | --- | --- |
| alpha-S1-casein | Pepsin (pH > 2) | MK |
|  |  | LI |
|  |  | TC |
|  |  | VAVA |
|  |  | ARPKY* |
|  |  | PLRY |
|  |  | PEV |
|  |  | QNEPDSIEEV |
|  |  | NKRKILE |
|  |  | AVVSPIQ |
|  |  | RQENIDE |
|  |  | KDTRNEPTEDHIMEDTERKESGSSSSEEVVSSTTEQKDIL |
|  |  | KEDMPSQRY |
|  |  | LEE |
|  |  | HRLNK |
|  |  | KL |
|  |  | LQ |
|  |  | EAIRDQK |
|  |  | LIPRVKL* |
|  |  | SSHP |
|  |  | YL |
|  |  | EQ |
|  |  | RINEDNHPQL |
|  |  | GEPVKVVTQEQA |
|  |  | LEPFPQF |
|  |  | GASP |
|  |  | YVA |
|  |  | YYPPQVMQ |
|  |  | IAHPSS |
|  |  | DTPEGIASEDGGKTDVMPQW |
|  | Trypsin | MK |
|  |  | LLILTCLVAVALARPK |
|  |  | YPLR |
|  |  | YPEVFQNEPDSIEEVLNK |
|  |  | ILELAVVSPIQFR |
|  |  | QENIDELK |
|  |  | DTR |
|  |  | NEPTEDHIMEDTER |
|  |  | ESGSSSSEEVVSSTTEQK |
|  |  | DILK |
|  |  | EDMPSQR |
|  |  | YLEELHR |
|  |  | LNK |
|  |  | YK |
|  |  | LLQLEAIR |
|  |  | DQK |
|  |  | LIPR |
|  |  | VK |
|  |  | LSSHPYLEQLYR |
|  |  | INEDNHPQLGEPVK |
|  |  | VVTQEQAYFHLEPFPQFFQLGASPYVAWYYPPQVMQYIAHPSSYDTPEGIASEDGGK |
|  |  | TDVMPQWW |
| alpha-S2-casein | Pepsin (pH > 2) | MK |
|  |  | FI |
|  |  | TC |
|  |  | AVV |
|  |  | AKHEMDQGSSSEESINVSQQK |
|  |  | KQVKKVAIHPSKEDICST |
|  |  | CEEAVRNIKEVESAEVPTENKISQ |
|  |  | QK |
|  |  | KF |
|  |  | LQ |
|  |  | QA |
|  |  | HQGQIVMNP |
|  |  | WDQGKTRAYP |
|  |  | FIPTVNTEQ |
|  |  | SISEESTEVPTEESTEV |
|  |  | TKKTEL |
|  |  | TEEEKDHQKF |
|  |  | LNKI |
|  |  | YQ |
|  |  | QT |
|  |  | LW |
|  |  | PEY |
|  |  | KTVY |
|  |  | QKTMTP |
|  |  | WNHIKRYF* |
|  | Trypsin | MK |
|  |  | FFIFTCLLAVVLAK* |
|  |  | HEMDQGSSSEESINVSQQK |
|  |  | FK |
|  |  | QVK |
|  |  | VAIHPSK |
|  |  | EDICSTFCEEAVR |
|  |  | NIK |
|  |  | EVESAEVPTENK |
|  |  | ISQFYQK |
|  |  | WK |
|  |  | FLQYLQALHQGQIVMNPWDQGK |
|  |  | TR |
|  |  | AYPFIPTVNTEQLSISEESTEVPTEESTEVFTK |
|  |  | TELTEEEK |
|  |  | DHQK |
|  |  | FLNK |
|  |  | IYQYYQTFLWPEYLK |
|  |  | TVYQYQK |
|  |  | TMTPWNHIK |
|  |  | YF |
| beta-casein | Pepsin (pH > 2) | MKV |
|  |  | LI |
|  |  | ACRVAL |
|  |  | AREKEEF |
|  |  | KTAGEA |
|  |  | ESISSSEESITHINKQKIEK |
|  |  | KIEEQQQTEDEQQDKI |
|  |  | YTF |
|  |  | PQPQS |
|  |  | SHTEPIPYPIL |
|  |  | PQN |
|  |  | FLPPLQPAVMVP |
|  |  | FLQPKVMDVPKTKETIIPKRKEMP |
|  |  | LL |
|  |  | QSPVVP |
|  |  | FTESQS |
|  |  | TD |
|  |  | EN |
|  |  | HL |
|  |  | PLP |
|  |  | LL |
|  |  | QS |
|  |  | QIPQPVPQTPMIPPQS |
|  |  | SQ |
|  |  | KVLPVPQQMVPYPQRAMPVQAVL |
|  |  | FQEPVPDPVRG |
|  |  | LHPVPQP |
|  |  | LVPVIA |
|  | Trypsin | MK |
|  |  | VLILACR |
|  |  | VALALAR |
|  |  | EK |
|  |  | EEFK |
|  |  | TAGEALESISSSEESITHINK |
|  |  | QK |
|  |  | IEK |
|  |  | FK |
|  |  | IEEQQQTEDEQQDK |
|  |  | IYTFPQPQSLVYSHTEPIPYPILPQNFLPPLQPAVMVPFLQPK |
|  |  | VMDVPK |
|  |  | TK |
|  |  | ETIIPK |
|  |  | EMPLLQSPVVPFTESQSLTLTDLENLHLPLPLLQSLMYQIPQPVPQTPMIPPQSLLSLS |
|  |  | QFK |
|  |  | VLPVPQQMVPYPQR |
|  |  | AMPVQAVLPFQEPVPDPVR |
|  |  | GLHPVPQPLVPVIA |
| kappa-casein | Pepsin (pH > 2) | MKS |
|  |  | FF |
|  |  | VVTI |
|  |  | TL |
|  |  | FL |
|  |  | GAEVQNQEQPTCCEKVERL |
|  |  | LNEKTVK |
|  |  | YFPIQ |
|  |  | VQSRY |
|  |  | PSY |
|  |  | GIN |
|  |  | QHRLAVPINNQ |
|  |  | FIPYPNY |
|  |  | AKPVAIRL |
|  |  | HAQIPQCQAL |
|  |  | PNIDPPTVERRPRPRPSF |
|  |  | IAIPPKKTQDKTVNPAINTVATVEPPVIPTAEPAVNTVVIAEASSE |
|  |  | ITTSTPETTTVQITSTEI |
|  | Trypsin | MK |
|  |  | SFFLVVTILALTLPFLGAEVQNQEQPTCCEK |
|  |  | VER |
|  |  | LLNEK |
|  |  | TVK |
|  |  | YFPIQFVQSR |
|  |  | YPSYGINYYQHR |
|  |  | LAVPINNQFIPYPNYAKPVAIR |
|  |  | LHAQIPQCQALPNIDPPTVER |
|  |  | RPRPRPSFIAIPPK |
|  |  | TQDK |
|  |  | TVNPAINTVATVEPPVIPTAEPAVNTVVIAEASSEFITTSTPETTTVQITSTEI |
| albumin | Pepsin (pH > 2) | MK |
|  |  | VT |
|  |  | IS |
|  |  | SSV |
|  |  | SRGVF |
|  |  | RRDTHKSEIAHRFKD |
|  |  | LGEDD |
|  |  | KG |
|  |  | LV |
|  |  | IA |
|  |  | SQ |
|  |  | QQCP |
|  |  | FDDHVKL |
|  |  | VNEVTE |
|  |  | AKTCVADESAADCDKS |
|  |  | LHT |
|  |  | LF |
|  |  | GDK |
|  |  | CTVAS |
|  |  | RETY |
|  |  | GEMADCCEKQEPERNEC |
|  |  | QHKSDNPDL |
|  |  | PKLKPEPEA |
|  |  | CTA |
|  |  | QENEKRFGGK |
|  |  | LY |
|  |  | EIARRHPYF |
|  |  | YAPEL |
|  |  | AHQ |
|  |  | YKHVFEECCKDADKAAC |
|  |  | LL |
|  |  | PKL |
|  |  | DA |
|  |  | KERI |
|  |  | LASSARQRL |
|  |  | RCTSIQK |
|  |  | GDRA |
|  |  | LKA |
|  |  | WSVGH* |
|  |  | SQKF |
|  |  | PKADF |
|  |  | AEISKIVTD |
|  |  | TKIHKECCQGD |
|  |  | ECADDRADL |
|  |  | AK |
|  |  | FCDNQETISSK |
|  |  | KECCEKP |
|  |  | LL |
|  |  | EKSHCIHEAERDEMPENL |
|  |  | PAITEQ |
|  |  | AEDKDVCKH |
|  |  | YTEEKDVF |
|  |  | GM |
|  |  | HE |
|  |  | YARRHPEY |
|  |  | AVS |
|  |  | RIAKE |
|  |  | YEAT |
|  |  | EDCCAKDDPHACY |
|  |  | ATV |
|  |  | DK |
|  |  | QH |
|  |  | ADEPQN |
|  |  | VKQNCE |
|  |  | EK |
|  |  | GE |
|  |  | QNDI |
|  |  | VRY |
|  |  | TKRLPQVSTPTL |
|  |  | VEVARG |
|  |  | LGRVGTKCCTL* |
|  |  | PESNRMSCAED |
|  |  | NRL |
|  |  | CV |
|  |  | HEKTPVSPRVTKCCTES |
|  |  | VNRRPCF |
|  |  | SS |
|  |  | TADET |
|  |  | YEPKE |
|  |  | FDEKT |
|  |  | FT |
|  |  | HADL |
|  |  | CSVSEPEKQIKKQTA |
|  |  | AE |
|  |  | KHKPKATDEQ |
|  |  | KTVMEK |
|  |  | VA |
|  |  | VDKCCAAVDKEAC |
|  |  | TVEV |
|  |  | VC |
|  |  | LIP |
|  |  | FAVA |
|  |  | DQARD |
|  | Trypsin | MK |
|  |  | WVTFISLLFLFSSVYSR |
|  |  | GVFR |
|  |  | DTHK |
|  |  | SEIAHR |
|  |  | FK |
|  |  | DLGEDDFK |
|  |  | GLVLIAFSQYLQQCPFDDHVK |
|  |  | LVNEVTEFAK |
|  |  | TCVADESAADCDK |
|  |  | SLHTLFGDK |
|  |  | LCTVASLR |
|  |  | ETYGEMADCCEK |
|  |  | QEPER |
|  |  | NECFLQHK |
|  |  | SDNPDLPK |
|  |  | LKPEPEALCTAFQENEK |
|  |  | FGGK |
|  |  | YLYEIAR |
|  |  | RHPYFYAPELLYYAHQYK |
|  |  | HVFEECCKDADK |
|  |  | AACLLPK* |
|  |  | LDALK |
|  |  | ER |
|  |  | ILASSAR |
|  |  | QR |
|  |  | LR |
|  |  | CTSIQK |
|  |  | FGDR |
|  |  | ALK |
|  |  | AWSVGHLSQK |
|  |  | FPK |
|  |  | ADFAEISK |
|  |  | IVTDLTK |
|  |  | IHK |
|  |  | ECCQGDLLECADDR |
|  |  | ADLAK |
|  |  | YFCDNQETISSK |
|  |  | LK |
|  |  | ECCEKPLLEK |
|  |  | SHCIHEAER |
|  |  | DEMPENLPAITEQFAEDKDVCKHYTEEK |
|  |  | DVFLGMFLHEYAR |
|  |  | RHPEYAVSLLLR |
|  |  | IAK |
|  |  | EYEATLEDCCAK |
|  |  | DDPHACYATVFDK |
|  |  | LQHLADEPQNLVK |
|  |  | QNCELFEK |
|  |  | LGEYGFQNDILVR |
|  |  | YTK |
|  |  | LPQVSTPTLVEVAR |
|  |  | GLGR |
|  |  | VGTK |
|  |  | CCTLPESNR |
|  |  | MSCAEDYLSLILNR |
|  |  | LCVLHEK |
|  |  | TPVSPR |
|  |  | VTK |
|  |  | CCTESLVNR |
|  |  | RPCFSSLTADETYEPK |
|  |  | EFDEK |
|  |  | TFTFHADLCSVSEPEK |
|  |  | QIK |
|  |  | QTALAELLK |
|  |  | HKPK |
|  |  | ATDEQLK |
|  |  | TVMEK |
|  |  | FVAFVDK |
|  |  | CCAAVDK |
|  |  | EACFTVEVLALFVFMFVCLIPFAVAFDQAR |
| alpha-lactalbumin | Pepsin (pH > 2) | MMS |
|  |  | VS |
|  |  | VGI |
|  |  | LF |
|  |  | PTIQAKQ |
|  |  | FTKCKL* |
|  |  | SDE |
|  |  | KDMNGHGGIT |
|  |  | AE |
|  |  | ICII |
|  |  | HMSG |
|  |  | DTETVVSNNGNRE |
|  |  | YG |
|  |  | QINNKI |
|  |  | WCRDNEN |
|  |  | QSRNICDISCDK |
|  |  | LDDD |
|  |  | TDDKMCAKKILDKEGID |
|  |  | AHKPLCSEK |
|  |  | EQ |
|  |  | QCEK |
|  | Trypsin | MMSLVSLLLVGILFPTIQAK |
|  |  | QFTK |
|  |  | CK |
|  |  | LSDELK |
|  |  | DMNGHGGITLAEWICIIFHMSGYDTETVVSNNGNR |
|  |  | EYGLFQINNK |
|  |  | IWCR |
|  |  | DNENLQSR |
|  |  | NICDISCDK |
|  |  | FLDDDLTDDK |
|  |  | MCAK |
|  |  | ILDK |
|  |  | EGIDYWLAHKPLCSEK |
|  |  | LEQWQCEK |
| * The peptides with both anticancer and antimicrobial activity and having high stability and half-life in intestine environment. | | |

**Table S2. The binding energy and inhibition constant (Ki) of the interaction of desired peptides and various receptors using molecular docking**

| **Receptor** | **Peptide** | **Binding energy (Kcal/mol)** | **Inhibition constant (μM)** |
| --- | --- | --- | --- |
| ERα | P1 | - 6.0 | 58.9 |
|  | P2 | - 5.3 | 184.1 |
|  | **P3** | **- 7.1** | **9.9** |
|  | P4 | - 5.9 | 69.8 |
|  | P5 | - 7.0 | 11.8 |
|  | P6 | - 5.5 | 133.7 |
|  | P7 | - 4.9 | 352.7 |
|  | P8 | - 6.1 | 50.2 |
| MCL-1 | P1 | - 6.2 | 42.8 |
|  | P2 | - 5.5 | 133.7 |
|  | **P3** | **- 7.2** | **8.5** |
|  | P4 | - 6.5 | 26.5 |
|  | P5 | - 6.2 | 42.8 |
|  | P6 | - 5.5 | 133.7 |
|  | P7 | - 4.7 | 490.5 |
|  | P8 | - 6.3 | 36.4 |
| PBP1a | P1 | - 6.9 | 13.8 |
|  | P2 | - 5.9 | 69.8 |
|  | P3 | - 6.8 | 16.2 |
|  | P4 | - 7.0 | 11.8 |
|  | **P5** | **- 7.5** | **5.2** |
|  | P6 | - 6.0 | 59.5 |
|  | P7 | - 5.8 | 81.9 |
|  | P8 | - 6.3 | 36.4 |
| IARS | P1 | - 7.6 | 4.4 |
|  | P2 | - 6.8 | 16.2 |
|  | **P3** | **- 8.4** | **1.2** |
|  | P4 | - 7.5 | 5.2 |
|  | P5 | - 7.8 | 3.2 |
|  | P6 | - 7.1 | 9.9 |
|  | P7 | - 7.0 | 11.8 |
|  | P8 | - 7.5 | 5.2 |
| DNA gyrase | P1 | - 5.7 | 97.1 |
|  | P2 | - 5.9 | 69.8 |
|  | P3 | - 6.4 | 31.0 |
|  | **P4** | **- 7.0** | **11.8** |
|  | P5 | - 6.6 | 22.5 |
|  | P6 | - 5.5 | 133.7 |
|  | P7 | - 4.9 | 352.7 |
|  | P8 | - 5.7 | 97.1 |
| DHFR | P1 | - 7.0 | 11.8 |
|  | P2 | - 6.4 | 31.0 |
|  | P3 | - 7.8 | 3.2 |
|  | P4 | - 6.6 | 22.5 |
|  | **P5** | **- 8.7** | **0.74** |
|  | P6 | -6.3 | 36.4 |
|  | P7 | - 6.4 | 31.0 |
|  | P8 | - 6.9 | 13.8 |
| ***Note:*** Data are expressed as mean of three independent runs. | | | |


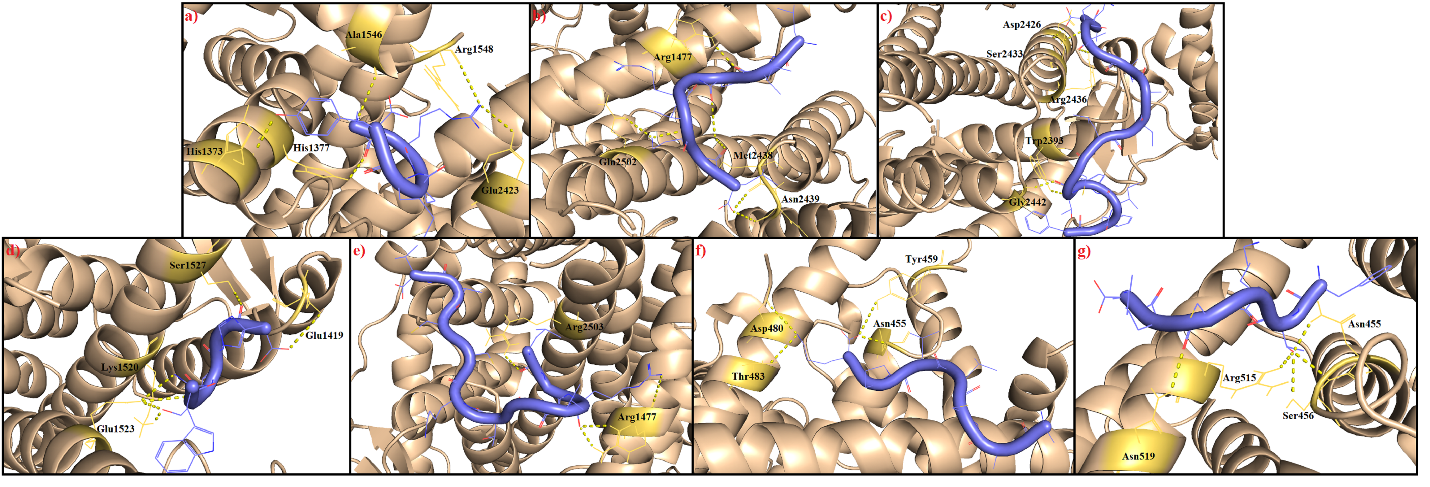


**Fig S1.** **Molecular interactions between various peptides and ERα using molecular docking analysis. a) P1-ERα complex, b) P2-ERα complex, c) P4-ERα complex, d) P5-ERα complex, e) P6-ERα complex, f) P7-ERα complex, g) P8-ERα complex. All hydrogen bonds have shown in dashed lines with yellow color.**


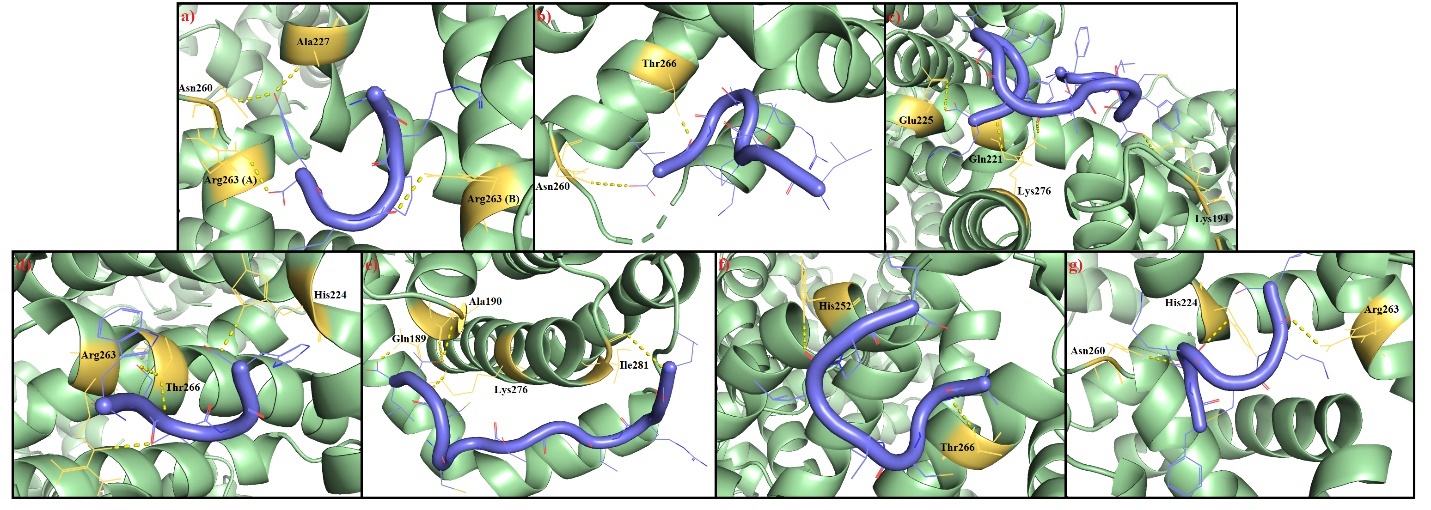


**Fig S2.** **Molecular interactions between various peptides and MCL-1 using molecular docking analysis. a) P1-MCL-1 complex, b) P2-MCL-1 complex, c) P4-MCL-1 complex, d) P5-MCL-1 complex, e) P6-MCL-1 complex, f) P7-MCL-1 complex, g) P8-MCL-1 complex. All hydrogen bonds have shown in dashed lines with yellow color.**


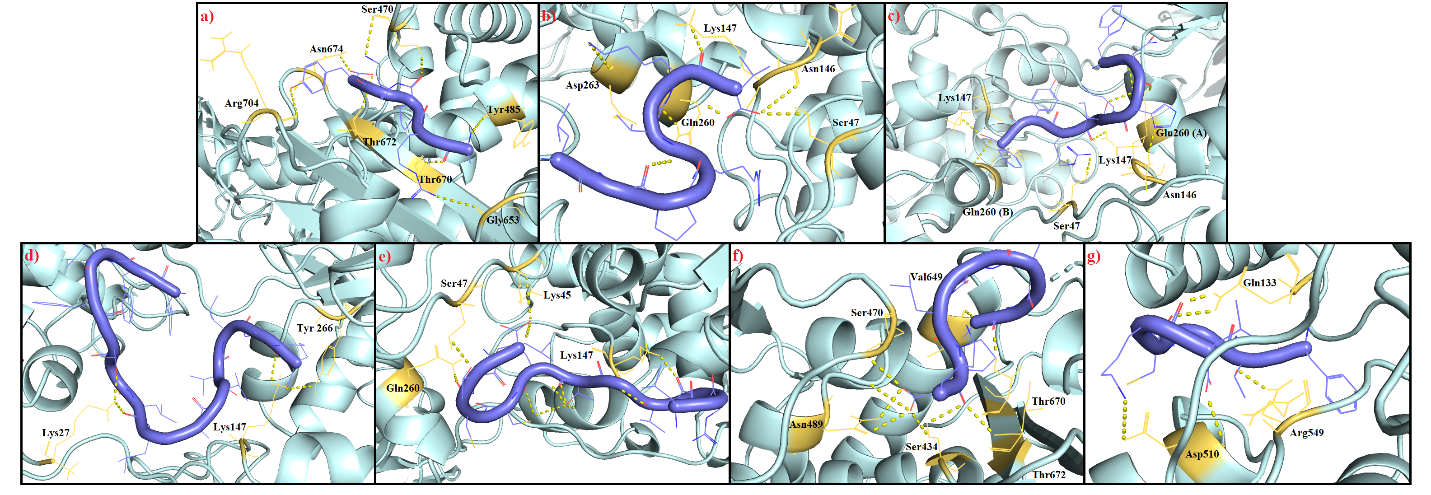


**Fig S3.** **Molecular interactions between various peptides and PBP1a using molecular docking analysis. a) P1- PBP1a complex, b) P2- PBP1a complex, c) P3- PBP1a complex, d) P4- PBP1a complex, e) P6- PBP1a complex, f) P7- PBP1a complex, g) P8- PBP1a complex. All hydrogen bonds have shown in dashed lines with yellow color.**


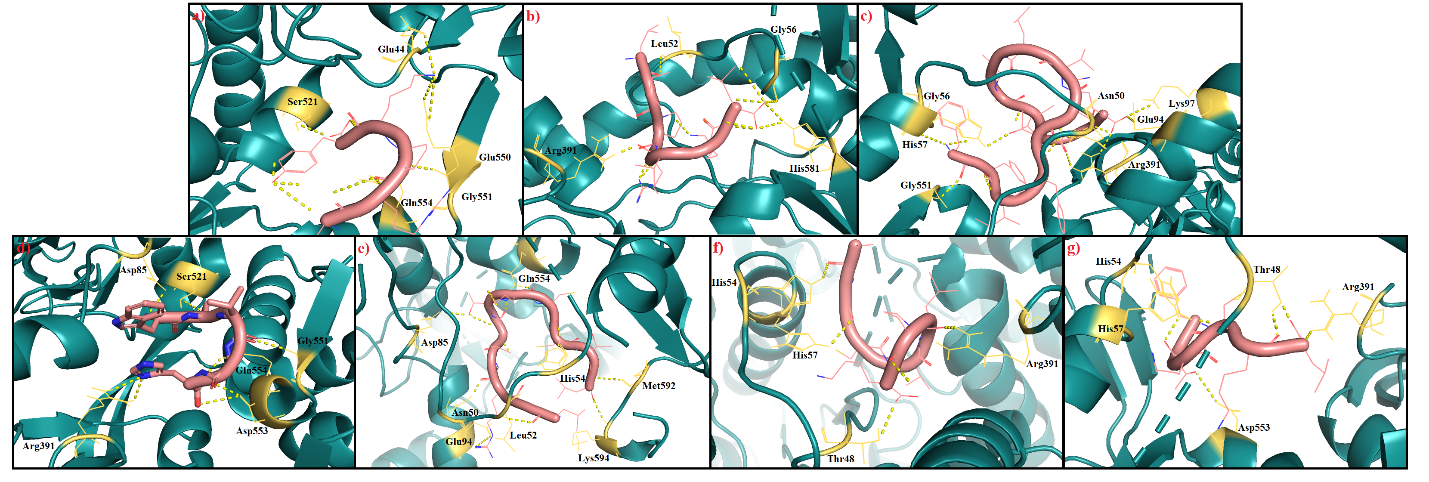


**Fig S4.** **Molecular interactions between various peptides and IARS using molecular docking analysis. a) P1-IARS complex, b) P2-IARS complex, c) P4-IARS complex, d) P4-IARS complex, e) P6-IARS complex, f) P7-IARS complex, g) P8-IARS complex. All hydrogen bonds have shown in dashed lines with yellow color.**


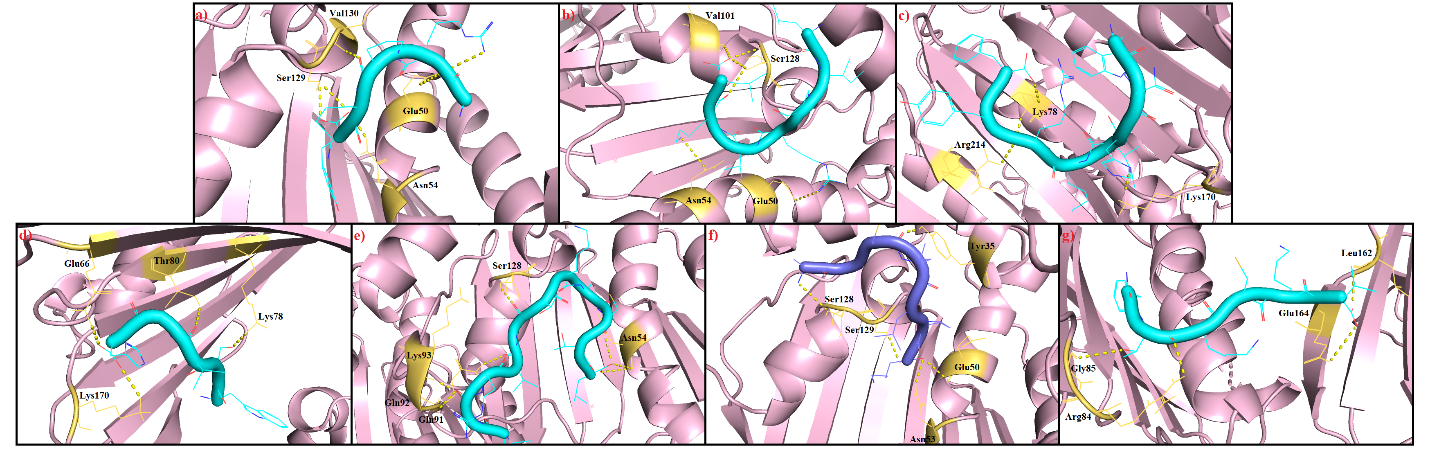


**Fig S5.** **Molecular interactions between various peptides and DNA gyrase using molecular docking analysis. a) P1-DNA gyrase complex, b) P2-DNA gyrase complex, c) P3-DNA gyrase complex, d) P5-DNA gyrase complex, e) P6-DNA gyrase complex, f) P7-DNA gyrase complex, g) P8-DNA gyrase complex. All hydrogen bonds have shown in dashed lines with yellow color.**


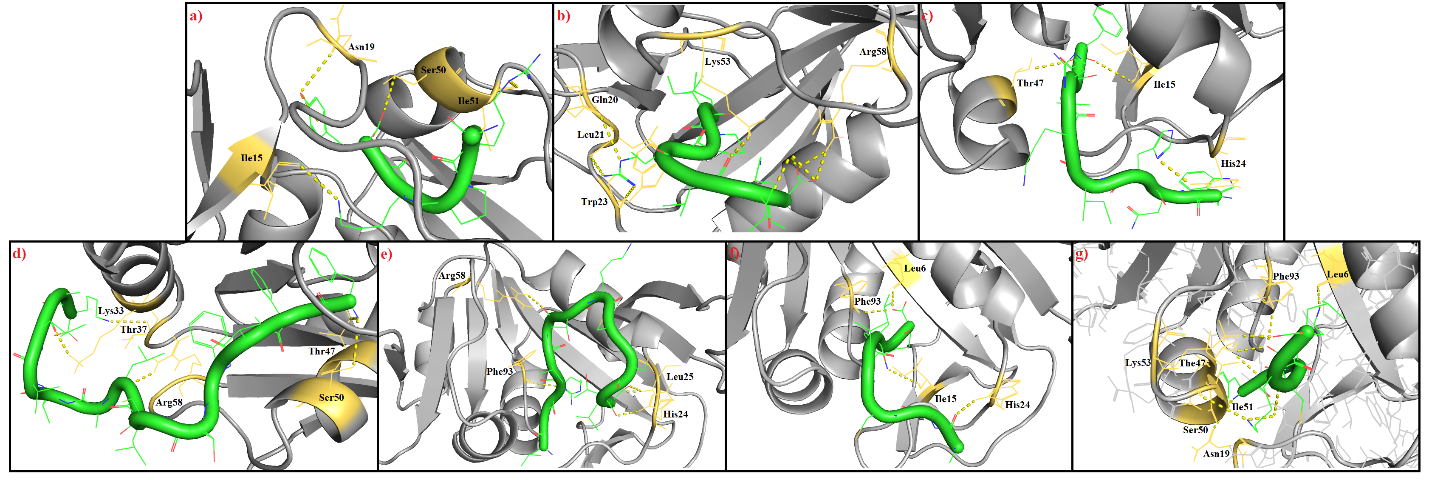


**Fig S6.** **Molecular interactions between various peptides and DHFR using molecular docking analysis. a) P1-DHFR complex, b) P2-DHFR complex, c) P3-DHFR complex, d) P5-DHFR complex, e) P6-DHFR complex, f) P7-DHFR complex, g) P8-DHFR complex. All hydrogen bonds have shown in dashed lines with yellow color.**
