# Supplementary material for: In vitro and in silico studies for the identification of anti-cancer and antibacterial peptides from camel milk protein hydrolysates
Source: PLoS One. 2023 Jul 12;18(7):e0288260. doi: 10.1371/journal.pone.0288260 (PMC10337890; doi:10.1371/journal.pone.0288260)
Supplement: Attachment — Submitted filename: Response to Reviewers.docx [file pone.0288260.s002.docx]

Reviewer #1: Minor modifications required:

1. Indicate the bioactivities that are being investigated in the study in your title - i.e. you can add anti-cancer and anti-bacterial bioactivities in the title.

**Response: Dear Reviewer, we would like to thank you for your time and for helping improve our manuscript with your valuable comments.**

**Title was revised as you wished.**

2. Line 43: Remove the link and reference it according to the guidelines.

**Response: we revised it according to the journal guideline.**

3. Line 80: State the storage conditions between the period of purchase and analysis.

**Response: we added it in the main text (line 81).**

4. Line 90: Avoid using ddH2O, instead use distilled water/double distilled water accordingly.

**Response: we revised it as you wished.**

5. Table 4 in the reference "<https://doi.org/10.1016/j.foodchem.2021.131444>" has a list of camel milk-derived bioactive peptides with potential antibacterial properties. Check it out and use it in your discussion to see if there are any similar trends.

**Response: Thank you for your good suggestion. We included it in the discussion (lines 221-228).**

Reviewer #2: The manuscript "In vitro and in silico studies for identification bioactive peptides from camel milk protein hydrolysates" is a manuscript that investigates isolating the protein from camel milk and hydrolyzing it using two different enzymes to achieve the hydrolysate fractions with biological activities. In my opinion, the study has been well-designed, and the manuscript covers the objectives. However, some points should be carefully addressed by the authors. I believe this manuscript needs MAJOR Modification to be suitable for publishing in the journal.

Comments:

1. Title: Add "the" and "of" to the title. In vitro and in silico studies for the identification of bioactive peptides from camel milk protein hydrolysates

**Response:** **Dear Reviewer, we would like to thank you for your time and for helping improve our manuscript with your valuable comments.**

**Title was edited as you wished.**

2. Introduction- Page 9, Line 43: use the references according to the journal requirement for web pages.

**Response: we revised it according to the journal guideline.**

3. Materials and Methods- Page 11, Line 93: Why did the authors select 12 h for hydrolysis of the proteins?

**Response: This time was chosen due to (i) Ensure the complete hydrolysis of the protein by the enzyme, that is, the enzyme has the opportunity to recognize and cut all its recognition sites (ii) Since our aim was to evaluate the antibacterial and anticancer activities, therefore we wanted to ensure the production of 1-7 kD peptides because the peptides with these sizes usually exhibit these activities (**[**https://doi.org/10.1098/rsob.200004**](https://doi.org/10.1098/rsob.200004)**; https://doi.org/ 10.1128/AAC.02369-13). Also, in a study by Wang et al. (https://doi.org/10.3390/ani10020337), they stated that hydrolysis of camel milk proteins for 12h could produce peptides for killing their desired bacteria (lines 228-232).**

4. Did the authors measure the DH of the parent protein as a control? If not, how do they justify that the DH of protein hydrolysates is higher or lower than the parent protein?

**Response: Thank you for your precious question. At time 0,** **no hydrolysis has taken place, so this in turn represents the degree of hydrolysis of the original proteins initially. However, we added the following sentence: “At time 0, no hydrolysis has taken place, so that the degree of hydrolysis of the parent proteins varied from 1.9 to 2.8% and increased over time with the addition of enzymes (Fig. 1)" in lines 195-197 to avoid any misunderstanding.**

5. The above question is important about the bioactivities of the protein hydrolysates. How do they claim that the anticancer activity of protein hydrolysates is higher than the parent proteins? Please justify.

**Response: According to the Fig. 2,** **we measured anticancer activity of parent proteins and hydrolysates, therefore whey and casein proteins (parent proteins that used as control) respectively killed approximately 30% and 10% of total cancerous cells, which is lower than anticancer activity of hydrolysates (Fig. 2). Also, it is better to say that since we boiled the mixture after hydrolysis to inactivate the enzyme, all of proteins, including enzyme and parent proteins were removed using centrifugation. Therefore, there are only peptides in the mixture. But if there are parent proteins in the mixture, they are inactive. However, we clarified it by adding a sentence in lines 250-252.**

6. Why do authors use only 4 bacteria for the antibacterial activities? For antibacterial studies, usually, a combination of gram-negative and gram-positive bacteria should be used. Justify please.

**Response: Thank you so much for your good question. As you said we also used a combination of gram-negative and gram-positive bacteria (3 gram-negatives bacteria, including *Klebsiella pneumoniae* (PTCC 1053), *Pseudomonas aeruginosa* (PTCC 1074), and *Escherichia coli* (PTCC 1330); and 1 gram-positive bacteria, including *Staphylococcus aureus* (PTCC 1112)). They are from different families and commonly use in antibacterial studies, because they cause deadly diseases in humans. Therefore, we decided to choose them for evaluating antibacterial tests.**
